# Improving biomedical entity linking for complex entity mentions with LLM-based text simplification

**DOI:** 10.1093/database/baae067

**Published:** 2024-07-26

**Authors:** Florian Borchert, Ignacio Llorca, Matthieu-P Schapranow

**Affiliations:** Hasso Plattner Institute for Digital Engineering, University of Potsdam, Prof.-Dr.-Helmert-Straße 2-3, Potsdam 14482, Germany; Hasso Plattner Institute for Digital Engineering, University of Potsdam, Prof.-Dr.-Helmert-Straße 2-3, Potsdam 14482, Germany; Hasso Plattner Institute for Digital Engineering, University of Potsdam, Prof.-Dr.-Helmert-Straße 2-3, Potsdam 14482, Germany

## Abstract

Large amounts of important medical information are captured in free-text documents in biomedical research and within healthcare systems, which can be made accessible through natural language processing (NLP). A key component in most biomedical NLP pipelines is entity linking, i.e. grounding textual mentions of named entities to a reference of medical concepts, usually derived from a terminology system, such as the Systematized Nomenclature of Medicine Clinical Terms. However, complex entity mentions, spanning multiple tokens, are notoriously hard to normalize due to the difficulty of finding appropriate candidate concepts. In this work, we propose an approach to preprocess such mentions for candidate generation, building upon recent advances in text simplification with generative large language models. We evaluate the feasibility of our method in the context of the entity linking track of the BioCreative VIII SympTEMIST shared task. We find that instructing the latest Generative Pre-trained Transformer model with a few-shot prompt for text simplification results in mention spans that are easier to normalize. Thus, we can improve recall during candidate generation by 2.9 percentage points compared to our baseline system, which achieved the best score in the original shared task evaluation. Furthermore, we show that this improvement in recall can be fully translated into top-1 accuracy through careful initialization of a subsequent reranking model. Our best system achieves an accuracy of 63.6% on the SympTEMIST test set. The proposed approach has been integrated into the open-source xMEN toolkit, which is available online via https://github.com/hpi-dhc/xmen.

## Introduction

Extraction and normalization of information captured in medical free-text documents are prerequisites for their use in research, clinical decision support, personalized medicine, and other downstream applications [1, 2]. An important task within biomedical information extraction pipelines is entity linking (EL) i.e. mapping mentions of named entities to their corresponding concepts in a structured knowledge base (KB), which is typically based on a controlled vocabulary or other medical terminology system [3].

The canonical architecture for EL comprises two main steps: candidate generation and candidate ranking [4]. Candidate generation aims to produce a list of potential concept matches for a given mention, while the ranking step prioritizes these candidates based on relevance and context. Within the candidate generation phase, achieving high recall is essential: the goal is to ensure that the correct entity is included among the candidates generated for subsequent reranking. Failure to generate candidates accounts for a large fraction of errors in current biomedical EL systems [5].

Candidate generation becomes particularly challenging when there is a mismatch between KB aliases and entity mentions. Various strategies have been proposed for improving performance in such cases, e.g. by obtaining additional (multi-lingual) KB aliases [6] or using coreference information across documents [7]. An area that has received comparatively little attention is the handling of complex entity mentions, i.e. mentions spanning multiple tokens. Several recently released biomedical corpora feature a large proportion of such long mention spans, resulting from their specific annotation policies. Notable examples include not only the DisTEMIST [8] or SympTEMIST [9] corpora for the Spanish language but also GGPONC for German [10]. Complex entity mentions pose significant challenges for candidate generation algorithms that rely on similarity-based lookups (utilizing either dense or shallow representations) between the mention and potential candidates.

Recently, generative large language models (LLMs) have demonstrated their potential across a wide range of biomedical natural language processing (NLP) tasks, including text simplification and summarization [11]. However, for information extraction problems, such as named entity recognition and EL, the zero-shot and few-shot performance of general-purpose LLMs still falls short of the (supervised) state-of-the-art performance by a substantial margin [12]. More successful generative approaches for biomedical EL are instead based on sequence-to-sequence models, which perform well on English-language benchmark datasets but rely on language resources, such as pretrained encoder–decoder models and KB aliases, which might not be available for other languages [13, 14]. In contrast, generative biomedical EL approaches for non-English languages have received comparatively little attention. Zhu *et al*. [15] propose a prompt-based contrastive generation framework for biomedical EL in multiple languages. However, we note that the only gold-standard dataset used in their evaluations are two subsets of the MantraGSC [16], with reported performance substantially below the state-of-the-art for this dataset, following more classical generate-and-rank approaches [17].

**Figure 1.**
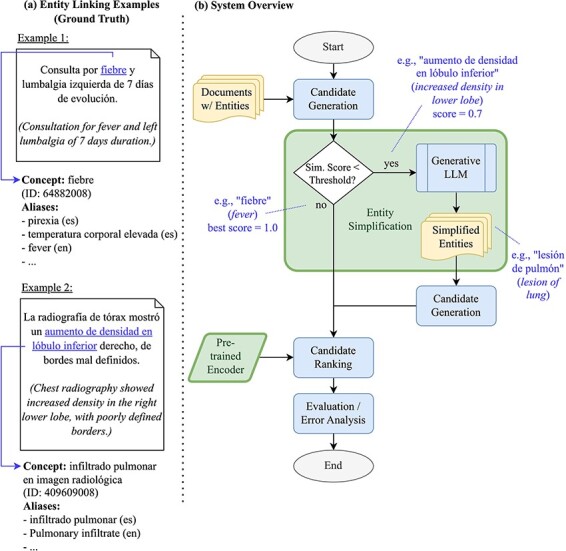
(a) Examples of entities and their grounding against SNOMED CT, as well as (b) an overview of our EL pipeline as a flow chart- the components investigated in this work are highlighted.

In this work, we investigate how LLMs can improve candidate generation performance for biomedical EL within a generate-and-rank framework. In particular, we employ LLMs to simplify mention spans, i.e. rephrasing them in a way that is more suitable for similarity search over concept aliases. Such simplification can take various forms: on the level of surface forms, it usually means that long mention spans are shortened. Syntactically and semantically, it might also involve converting adjectives to noun phrases, or choosing more general terms instead of specific ones. Our work builds upon our successful participation in the BioCreative VIII SympTEMIST shared task [9, 18]. The shared task provided a benchmark dataset of clinical case reports in Spanish with gold-standard annotations of symptom mentions and corresponding Systematized Nomenclature of Medicine Clinical Terms (SNOMED CT) codes [19]. Our winning submission employed the default EL pipeline implemented in the xMEN toolkit for cross-lingual medical entity normalization [17]. In this paper, we will share how the candidate generation phase of the system can be enhanced further through a reusable LLM-based entity simplification approach. The approach shares some similarities to Roller *et al*. [20], who translate non-English biomedical entity mentions to English, thus facilitating lookup in an English-language KB. As the xMEN system already accounts for the language mismatch through dense, cross-lingual candidate retrieval, this work investigates the additional benefit of paraphrasing the input mentions in the target language. An overview of our EL pipeline as a flow chart, along with example mentions, is depicted in Fig. 1. The figure shows two examples of mentions: a simple one, which can be easily normalized (“fiebre”), and a more complex one that benefits from simplification (“aumento de densidad en lóbulo inferior” is simplified to the more general term “lesión de pulmón”). In addition to improved candidate generation, we will also investigate how initialization of a cross-encoder-based reranker from different BERT models affects EL performance and to what extent reranking is able to leverage the additionally retrieved candidates.

In summary, the contributions of this work are:

- an easily reusable, LLM-based entity simplification approach,
- an analysis of its feasibility and the effect of entity simplification on recall during candidate generation, as well as
- an evaluation of the impact of the initialization of cross-encoders for reranking on final entity linking accuracy.

The remainder of this work is structured as follows: we introduce the SympTEMIST benchmark dataset and our EL pipeline, including the novel entity simplification component. Afterward, we share our experimental results, followed by a discussion and error analysis. Finally, we summarize our findings and conclude with an outlook.

## Materials and methods

In this section, we describe our incorporated methods. The source code to reproduce our experimental results is available online (https://github.com/hpi-dhc/symptemist/). Unless otherwise stated, we use the default configurations of the xMEN toolkit [17].

### Dataset

The SympTEMIST shared task dataset encompasses *n* = 1000 manually annotated case reports in Spanish. Twenty-five percent (*n* = 250) of the documents have been used as a held-out test set in the shared task and were made available thereafter. The remaining documents (*n* = 750) are used as training data, out of which we sample 20% (*n* = 150) as an internal validation set for model selection. For integration with the xMEN framework, we implement a data loader for the dataset within the BigBIO [21] library. In total, the dataset contains more than 12 000 annotations of symptoms, signs, and findings. However, for the shared task, composite entity mentions, i.e. with multiple concepts per span, were excluded from the evaluation, leaving us with 11 124 annotations.

Mention spans in SympTEMIST tend to be long, in comparison to other corpora, as shown in Table 1.

**Table 1.** Dataset statistics of SympTEMIST (version as used in the entity linking track of the shared task without composite mentions) and comparable biomedical corpora (largest values are highlighted bold)

			Mention length		
Corpus	Documents	Mentions	Mean	Median	Max
SympTEMIST	1000	11 K	**3.56**	**2**	**36**
DisTEMIST	1000	11 K	3.27	2	28
Quaero	2508	16 K	1.40	1	15
MedMentions	4392	2327 K	1.40	1	19

Measured as the number of whitespace-separated tokens, the average mention in SympTEMIST has a length of 3.56 tokens, with a median of 2 tokens and a maximum length of 36 tokens. This is comparable to the DisTEMIST dataset (mean: 3.27, median: 2, max: 28) and much longer than in other biomedical EL benchmark datasets, such as Quaero (mean: 1.40, median: 1, max: 15) or MedMentions (mean: 1.40, median: 1, max: 19) [22, 23].

### Target knowledgebase

The set of target concepts for EL in SympTEMIST consists of 121 760 SNOMED CT codes as defined by a gazetteer of concepts related to a task-relevant subset of SNOMED CT [9]. Using the xMEN command-line interface, we extend the limited number of aliases for this defined set of concepts with all Spanish and English aliases from the Unified Medical Language System (version 2023AB), resulting in ∼1.08 m terms [24].

### Candidate generation

For candidate generation, we use the default pipeline from the xMEN toolkit, which consists of an ensemble of (i) a **term frequency–inverse document frequency** (TF-IDF) vectorizer over character 3-grams as well as (ii) a cross-lingual SapBERT model [25]. Candidate concepts for each mention are retrieved based on a *k*-nearest-neighbor search, with cosine similarity over mention and concept vectors. We retrieve *k* = 64 candidates, which are used as an input to the subsequent reranking. As only concepts that are retrieved at this point can be possibly correctly ranked, it is essential to achieve high recall@k during this phase.

### LLM-based entity simplification

To increase recall for complex entity mentions, which tend to have little lexical overlap with aliases in the target terminology, we simplify mentions using a generative LLM. In this work, we employ, Generative Pre-trained Transformer model (GPT-4) (gpt-4-0125-preview) through the OpenAI API [26], as a proof of concept. However, our approach can also be used with any other generative LLM.

**Figure 2.**
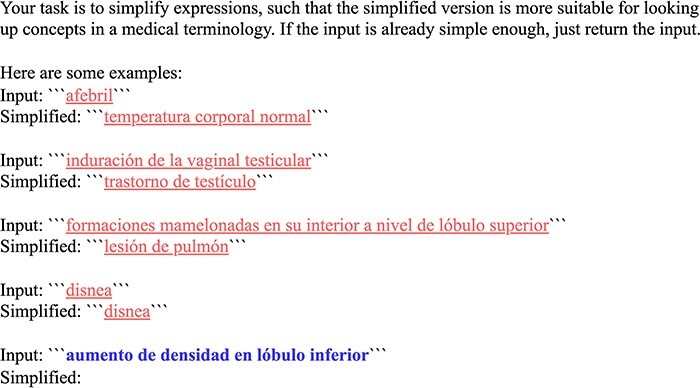
Few-shot prompt template for entity simplification: few-shot examples are underlined and the actual input mention is highlighted in bold, inputs and outputs are denoted by triple backticks (```).

We call the LLM using a few-shot prompt with four fixed in-context examples. Each example consists of a mention from the training set as well as the canonical concept name from the ground-truth concept in SNOMED CT. Figure 2 shows the used prompt template. Three few-shot examples were randomly sampled from the subset of mentions, for which the candidate generator fails to retrieve the ground-truth concept. This broad notion of simplification might also involve rewriting an adjective as a noun phrase (“afebril” as “temperature corporal normal”). One additional example has been chosen manually to demonstrate that easily linkable mentions (“disnea”) should not be further simplified.

However, it is not desirable to simplify all mentions, for instance, when candidates can already be generated with high confidence for the original mention span. We therefore consider a confidence threshold and apply mention simplification only when the top candidate concept for a mention has a score, i.e. cosine similarity, below this value, as shown in Fig. 1. For this subset of concepts, we apply text simplification and generate candidates again. The confidence threshold is a hyperparameter in the range [0, 1] that can be simply optimized by observing its impact on recall for the training set.

### Candidate ranking

The list of generated candidates is used as an input to a cross-encoder model for reranking [27]. The cross-encoder is trained for 20 epochs, using batches of 64 candidates with ground truth labels and a softmax loss with rank regularization [17]. We use the default hyperparameters of the xMEN library and keep the checkpoint that maximizes accuracy on the validation set. One training run takes ∼30 h on a single Nvidia A40 GPU. Since the main evaluation metric for the SympTEMIST shared task is accuracy (equivalent to recall@1), we do not consider a NIL (not-in-list) option during training and inference. This means a prediction is made for each mention and that recall is equal to precision or accuracy.

The cross-encoder model can be initialized from any pretrained BERT encoder. In our experiments, we investigate the initialization from the base-sized versions of the following models:

- **Multi-lingual, general-domain**: XLM-RoBERTa (FacebookAI/xlm-roberta-base), a multi-lingual model pretrained on general-domain text in 100 languages [28].
- **English, biomedical**: PubMedBERT (BiomedNLP-PubMedBERT-base-uncased-abstract-fulltext), pretrained on English biomedical text [29]. We include this state-of-the-art English model in our evaluation, as prior work has shown that such models can also outperform models for the specific target language for some tasks [30].
- **Spanish, general-domain**: BNE (roberta-base-bne), a RoBERTa model pretrained on a Spanish corpus from the National Library of Spain (Biblioteca Nacional de España) [31].
- **Spanish, biomedical**: A RoBERTa model (PlanTL-GOB-ES/roberta-base-biomedical-clinical-es) pretrained on Spanish biomedical text, which we refer to as PlanTL [32].

## Results

In the following, we share details about our conducted experiments and obtained results.

### Candidate generation with entity simplification

**Figure 3.**
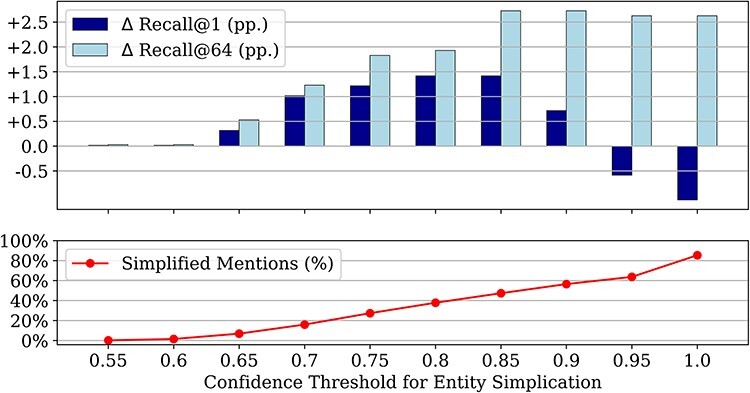
Impact of different confidence thresholds for entity simplification as the proportion of affected mentions out of all mentions, and the resulting improvements in recall@1 and recall@64 over the baseline candidate generator with the original mention spans (all results on the training set); we only simplify mentions when the baseline candidate generator produces a score below the threshold.

Figure 3 shows the impact of entity simplification on candidate generation on the training set for different confidence thresholds. The lowest score yielded by the baseline candidate generator is 0.55. Therefore, no simplification takes place below this threshold and thus the results are unaltered compared to the baseline. However, even for a threshold of 1.0, i.e. when all mentions are subject to simplification, some mentions remain unchanged, as the LLM may just return the original input when it cannot be further simplified.

The improvement in recall@64 over the baseline candidate generator increases up until a threshold of 0.85, then plateaus at a level of +2.9 pp. The difference in recall@1 also increases up until a threshold of 0.85, but then decreases and even becomes negative for too high thresholds. This highlights that the original candidate generator is highly confident about its top-1 choice for a subset of mentions, typically the ones with high lexical overlap between a mention and candidate alias. Altering such mentions by simplification is usually not helpful, and may indeed decrease recall@1 (while leaving recall@64 largely unaffected). As performance improvements are maximized for a threshold of 0.85, we use this threshold for all further experiments and test set evaluations.

Table 2 provides details on the impact of entity simplification during candidate generation (on the test set). Mentions are binned according to the maximum confidence score of the candidate generator. Confidence of 1.0 occurs when a direct lookup is possible, i.e. there is at least one exact match for an alias in the target KB. Results for simplification without a threshold, i.e. when all mentions are simplified, are reported for comparison (last column). As expected, the mean mention length is reduced, from 3.63 to 2.61 tokens. Comparing the confidence scores assigned by the candidate generator before and after simplification illustrates its effect: the number of mentions in the higher confidence brackets (>0.7) is reduced; at the same time, the number of mentions with lower candidate confidence scores increases. We interpret this change as the effect of generating more general terms for specific mentions, which have lower similarity with single (but potentially wrong) concepts in the candidate space. This way, relatively large increases in both recall@1 and recall@64 can be observed in the confidence range (0.6–0.9].

**Table 2.** Impact of entity simplification with a threshold of *t* = 0.85 by comparing test set performance before and after entity simplification, as well as the difference (Δ)

		No Simplification	Simplification (*t* = 0.85)	Δ	Simplification (*t* > 1.0)
Mention length	Max.	23	13	−10	13
	Mean	3.63	2.61	−1.02	2.83
Test set	Mentions	2847	2847		2847
	Recall@1	0.445	0.460	+0.015	0.431
	Recall@64	0.764	0.793	+0.029	0.792
Confidence score					
(0.5,0.6]	Mentions	37	85	+48	85
	Recall@1	0.027	0.048	+0.021	0.048
	Recall@64	0.243	0.241	−0.002	0.241
(0.6,0.7]	Mentions	444	616	+172	639
	Recall@1	0.065	0.096	+0.031	0.095
	Recall@64	0.338	0.481	+0.143	0.499
(0.7,0.8]	Mentions	653	577	−76	742
	Recall@1	0.219	0.328	+0.109	0.340
	Recall@64	0.629	0.744	+0.114	0.782
(0.8,0.9]	Mentions	555	411	−144	507
	Recall@1	0.438	0.494	+0.056	0.523
	Recall@64	0.845	0.910	+0.065	0.933
(0.9,1.0]	Mentions	433	433	±0	217
	Recall@1	0.718	0.718	±0	0.659
	Recall@64	0.977	0.977	±0	0.982
1.0	Mentions	725	725	±0	657
	Recall@1	0.756	0.756	±0	0.764
	Recall@64	0.986	0.986	±0	0.992

At the same time, there is no increase in the number of mentions linkable with very high confidence (>0.9) through simplification, i.e. none of the generated concepts is more suitable for a direct lexical lookup. Due to the applied threshold of 0.85, no reductions are visible in the higher confidence ranges as well. However, as shown in the last column of Table 2, and consistent with Fig. 3, simplifying entities with already high confidence scores would negatively impact the overall recall@1, highlighting the importance of selecting appropriate mentions for simplification.

### Impact of cross-encoder initialization on reranking performance

Table 3 shows the performance improvements on the test set, obtained through entity simplification during candidate generation (column “Simplification”) and reranking with different cross-encoder models (rows “Candidates + Reranking”). We report the recall@1, as accuracy was the main evaluation metric for the SympTEMIST shared task, which is equivalent to recall@1, given that no concept has multiple ground-truth codes. Recall@1 after candidate generation is improved by 1.5 pp via simplification on the test set, consistent with the training set performance shown in Fig. 3.

**Table 3.** Test set performance before and after entity simplification, with a threshold of *t* = 0.85 (highest values highlighted in bold, difference denoted by Δ)

				Accuracy/Recall@1 (test set)		
				No Simplification	Simplification (*t* = 0.85)	Δ
Candidates	xMEN Ensemble (TF-IDF + SapBERT)			0.445	0.460	+ 0.015
Candidates +Reranking (Cross-encoder)	XLM-RoBERTa	Multi-lingual	General	0.590	0.602	+ 0.012
	BNE	Spanish	General	0.581	0.595	+ 0.014
	PubMedBERT	English	Biomedical	0.579	0.585	+ 0.006
	PlanTL	Spanish	Biomedical	**0.607**	**0.636**	**+ 0.029**
Shared task	2nd Best Teams (BIT.UA, Fusion@SU)			0.589		

Subsequent training of a reranker with a Spanish biomedical model by PlanTL [32] benefits most from the additionally generated candidates, achieving an improvement of 2.9 pp in recall@1 over the baseline without simplification, which was the top-performing system in the original SympTEMIST shared task [18]. Strikingly, the model initialized from the PlanTL checkpoint can recover from nearly all ranking errors for the newly retrieved candidates, with the improvement in recall@1 of 2.9 pp being equal to the improvement in recall@64 before reranking. Considering the close performance of the top-performing teams in the shared task [33, 34], the magnitude of this effect is even more remarkable.

While the other settings for initializing the reranking model also benefit from improved candidate generation through entity simplification, their overall performance falls short of the Spanish biomedical model. Moreover, all improvements through simplification are already achieved after candidate generation, with no additional gains by reranking for these models.

## Discussion and evaluation

### Error analysis

**Figure 4.**
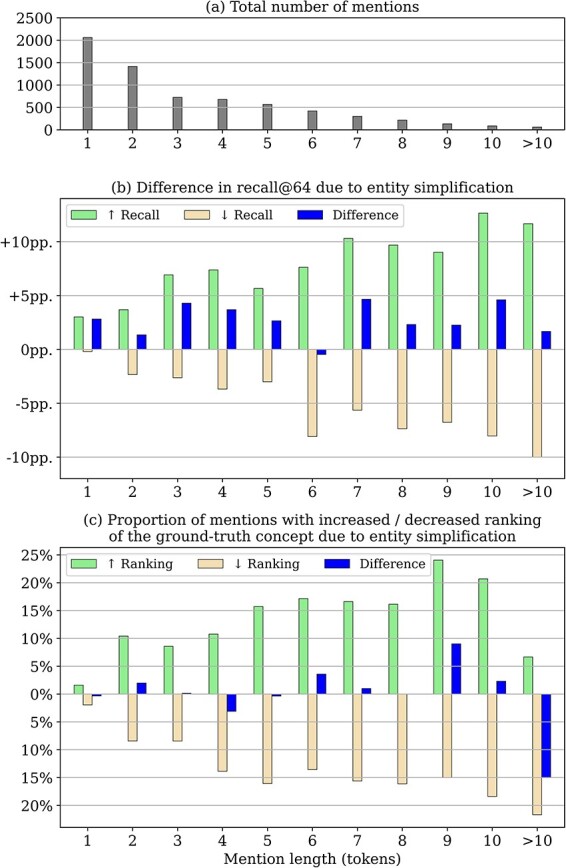
Impact of entity simplification for mentions of increasing length (in tokens, *x*-axis) - all results refer to the candidate generation phase (without reranking) on the training set: (a) total number of mentions by token length as context for the following relative measures (b) impact on overall recall@64, i.e. the fraction of mentions for which the correct candidate is retrieved after entity simplification versus the fraction of mentions where simplification is detrimental (c) fraction of mentions where simplification resulted in a higher or lower ranking of the correct concept.

Figure 4 shows a detailed analysis of the impact of entity simplification by mention length. The frequency of mentions of increasing lengths is shown in Fig. 4a. As shown in Fig. 4b, recall@64 is generally improved for mentions of all lengths, even for ∼3% of mentions of length 1. The analysis reveals that simplification can also lead to worse candidate generation performance, i.e. cases where the correct candidate is not among the top-64 candidates after simplification, although it was before simplification. However, the overall increase in recall for many mentions outweighs the decrease for others, which ultimately leads to the improved recall@64 as shown in Fig. 3.

In contrast, Fig. 4c shows the effect on the ranking of the correct concept for cases where it was already retrieved among the top-64 candidates. We can see that, on average, ranking performance is not affected in these instances. We note that there is a relatively large decrease for mentions of length 10 or more. However, the effect is negligible, as such mentions are very rare (see Fig. 4a).

The comparison of Fig. 4b and c shows that the improvement in recall@1 due to the simplification of the entities is based exclusively on the retrieval of additional, previously missed concepts and not on an improved ranking of candidates already among the top-64. We further conclude from Fig. 4b that the baseline candidate generation score is a helpful heuristic but not an exact criterion for identifying mentions that benefit most from entity simplification. A more accurate way to identify such mentions could potentially retain the achieved increase in recall, while avoiding most of the decrease.

### Limitations

Due to resource constraints, such as the daily token limits imposed by the OpenAI API, we were unable to perform any ablations on the used prompt. Therefore, a more refined prompt, i.e. with few-shot examples selected based on the respective input sentence, might yield improved results. To enable more exhaustive experiments in this regard, we plan to investigate the suitability of open-source LLMs in future work; however, currently substantial computational resources would also be required for hosting the most capable ones. In preliminary experiments, we briefly evaluated the open-source model BLOOMZ-7B [35] for entity simplification, but the output proved to be unreliable in comparison with GPT-4.

The English-language pretrained PubMedBERT model exhibits the worst performance across all reranking models, in particular in comparison to a Spanish-language, domain-specific model. Including PubMedBERT in our evaluation without any adaptation of the input text seemed plausible, due to its successful application for other non-English biomedical benchmark tasks and the similarity of medical terminology across languages [30]. However, the reranking step in our EL pipeline incorporates a high level of contextual information; therefore, models specifically trained to represent such context in the target language appear to be advantaged.

In addition, composite mentions (usually involving coordinate structures with “and”/“or”) have been excluded from the shared task evaluation. Not only do these tend to be complex but also present the additional difficulty of being mapped to multiple KB codes. While identifying and resolving such composite mentions might be achievable with generative LLMs as well, we have not investigated this issue further in the given work.

## Conclusion and outlook

In this work, we describe a novel technique for improving biomedical EL performance with text simplification in the context of the SympTEMIST shared task of the BioCreative VIII challenge. While the default EL pipeline of the incorporated open-source framework xMEN already achieved the best performance in the original shared task, we could show that the candidate generation and subsequent ranking performance for complex entity mentions can be further improved via LLM-based entity simplification with GPT-4 and a simple few-shot prompt, without elaborate prompt engineering.

We have integrated our proposed entity simplification approach as a new component into the modular architecture of the xMEN toolkit. Thus, it can be used with any EL benchmark dataset supported by the framework [17]. However, the complexity of manually or automatically annotated entity mention spans, which are subject to EL, strongly depends on the text genre and annotation policy of each dataset. An investigation into the applicability of our approach to other datasets, both with and without frequent complex mentions, is warranted. Similarly, the extension of our approach to composite mentions with multiple linked concepts remains open for future research.

The proposed approach can be extended along multiple dimensions. First, more extensive (automated) prompt engineering is likely to increase performance—for instance, it could provide benefits by dynamically selecting few-shot examples and including parts of the document context within the prompt. In the future, a more direct approach for using LLMs for biomedical EL could be achieved by enabling the generative LLM to access the target KB, for instance, via retrieval-augmented generation or function calling.

## Data Availability

The SympTEMIST dataset underlying this article are available on Zenodo, at https://zenodo.org/doi/10.5281/zenodo.8223653. The source code for our experiments is available on GitHub at https://github.com/hpi-dhc/symptemist.
